# Cytomegalovirus-Specific T Cells Persist at Very High Levels during Long-Term Antiretroviral Treatment of HIV Disease

**DOI:** 10.1371/journal.pone.0008886

**Published:** 2010-01-29

**Authors:** David M. Naeger, Jeffrey N. Martin, Elizabeth Sinclair, Peter W. Hunt, David R. Bangsberg, Frederick Hecht, Priscilla Hsue, Joseph M. McCune, Steven G. Deeks

**Affiliations:** 1 Department of Medicine, University of California San Francisco, San Francisco, California, United States of America; 2 Department of Epidemiology and Biostatistics, University of California San Francisco, San Francisco, California, United States of America; 3 Massachusetts General Hospital, Harvard Medical School, Harvard Initiative for Global Health, Boston, Massachusetts, United States of America; New York University, United States of America

## Abstract

**Background:**

In healthy, HIV seronegative, CMV seropositive adults, a large proportion of T cells are CMV-specific. High-level CMV-specific T cell responses are associated with accelerated immunologic aging (“immunosenesence”) in the elderly population. The impact of untreated and treated HIV infection on the frequency of these cells remains undefined.

**Methodology/Principal Findings:**

We measured the proportion of CD4+ and CD8+ T cells responding to CMV pp65 and IE proteins was measured using flow cytometry in 685 unique HIV seronegative and seropositive individuals. The proportion of CMV-specific CD8+ T cells was consistently higher in the HIV-seropositive subjects compared to the HIV-seronegative subjects. This HIV effect was observed even in patients who lacked measurable immunodeficiency. Among the HIV-seropositive subjects, CMV-specific CD8+ T cell responses were proportionately lower during recent infection, higher during chronic untreated infection and higher still during long-term antiretroviral treated infection. The CD8+ T cell response to just two CMV proteins (pp65 and IE) was approximately 6% during long-term therapy, which was over twice that seen in HIV-seronegative persons. CMV-specific CD4+ T cell responses followed the same trends, but the magnitude of the effect was smaller.

**Conclusions/Significance:**

Long-term successfully treated HIV infected patients have remarkably high levels of CMV-specific effector cells. These levels are similar to that observed in the elderly, but occur at much younger ages. Future studies should focus on defining the potential role of the CMV-specific inflammatory response in non-AIDS morbidity and mortality, including immunosenescence.

## Introduction

Cytomegalovirus (CMV) is a highly prevalent herpesvirus infection capable of causing end-organ disease in the setting of HIV-associated immunodeficiency. CMV infection has also been shown to be an independent factor associated with non-CMV end organ disease progression in untreated HIV disease [1], [2], [3], [4], [5], [6] and may negatively affect outcomes even in presence of successful antiretroviral therapy [7], [8]. Among a large cohort of treated patients, CMV viremia was associated with higher risk of mortality, independent of plasma HIV RNA level and CD4+ T cell counts [7]. Our group has recently shown that elevated CMV-specific immune responses are strongly associated with the level of atherosclerosis in HIV-infected, CMV-seropositive individuals [9], which may provide a mechanistic explanation for the consistent association between CMV and atherosclerosis in immunodeficient states [10].

There is also substantial evidence indicating that chronic CMV infection is associated with accelerated immunologic aging, or immunosenescence, in the elderly population [11], [12], [13], [14], [15] and in thymectomized younger adults [16]. Perhaps the strongest correlate of immunosenescence is the oligoclonal expansion of memory effector CD8+ T cells. These CD8+CD28− cells have limited ability to proliferate and are resistant to apoptosis [17], [18]. CMV-seropositive adults over the age of 65 to 70 have a much greater expansion of these CD28− cells than age-matched CMV-serogenegative controls (many of these cells reflect the oligoclonal expansion of CMV-specific T cells) [12], [19], [20], [21], [22], [23], [24]. The clinical significance of these findings is the focus of intense research; however, it has already been shown that CMV-seropositive older persons are less likely to respond to vaccines than age-matched, CMV-seronegative persons [13] and that CMV-associated changes in the immune system are highly predictive of early mortality among older persons [22], [25]. Finally, CMV seropositivity has been associated with higher rates of neurocognitive decline amongst the elderly [26], [27], while CMV reactivation in critically ill older patients predicts early mortality [15].

Despite the apparent importance of CMV infection in HIV infection, relatively little is known about CMV-specific T cell responses throughout the course of acute and chronic untreated disease, as well as during the course of long-term treated HIV disease. Methods have recently been developed and validated for high-throughput flow cytometric evaluation of antigen-specific T cell immune responses. When this approach was applied to a cohort of HIV seronegative CMV seropostive adults, it was demonstrated that approximately 5 to 10% of the circulating CD4+ and CD8+ T cell population recognize CMV in an antigen-specific manner [28]. A small subset of otherwise healthy adults may maintain over time an even higher levels of CMV-specific T cells [29]. Among HIV infected individuals, those on long-term HAART tend to have higher CMV-specific T cells responses then those who have progressive disease, or who have active CMV disease [28], [30], [31], [32], [33]. Long-term antiretroviral treated patients may also have higher levels of more differentiated CMV-specific CD8+ T cells than HIV negative individuals [34]. However, to our knowledge, no study has sought to assess in a comprehensive manner using large numbers of individuals the epidemiology of CMV-specific T cell immunity across the entire spectrum of HIV disease (including acute and chronic disease as well as untreated and treated disease). Also, no prior study was large enough to consider in the analysis other factors known to influence CMV specific immunity (e.g., age).

Here, we report a comprehensive survey of CMV-specific T cells in a large cohort of both HIV uninfected and infected adults. HIV-seropositive patients from all disease stages were selected, including those with acute disease, chronic antiretroviral untreated disease and long-term antiretroviral treated disease. For each of the analyses, we measured the proportion of freshly obtained CD4+ and CD8+ T cells that express high levels of interferon-γ in response to two of the most immunodominant CMV proteins [28]: pp65, a structural protein expressed throughout the viral life cycle, and the intermediate early (IE) protein.

## Methods

### Ethics Statement

This study was approved by the University of California, San Francisco Committee on Human Research (CHR). All participants provided written informed consent.

### Participants

HIV-infected participants were sampled from three prospective cohort studies in San Francisco. The SCOPE project is a prospective clinic-based cohort of chronically HIV-infected adults. T cell studies were performed consecutively in 395 participants. REACH is a community-based cohort of chronically HIV-infected adults. A subset of 60 consecutive participants consented to monthly adherence assessments and had immunophenotyping of T cells performed once during this time. The Options project is a cohort of individuals with evidence of acute or recent HIV-1 infection [35]. CMV specific T cell responses were performed in 147 consecutive participants who enrolled in the study. Subjects were stratified into four unique groups based on their clinical status at the time peripheral blood was obtained for this study: (1) recent untreated HIV infection (defined as having an estimated duration of infection of less that 180 days), (2) chronic untreated HIV infection, (3) chronic antiretroviral treated infection with undetectable plasma HIV RNA levels (<50 to 75 copies RNA/mL) and (4) chronic antiretroviral treated infection with detectable plasma HIV RNA levels.

As controls, HIV-uninfected but at-risk participants were sampled from a cohort of individuals who had received post-exposure prophylaxis following a sexual exposure to HIV. Forty-six HIV-seronegative individuals had immunophenotyping performed at a visit 48 weeks following their receipt of post-exposure prophylaxis. An additional 37 HIV-seronegative adults responded to poster and internet advertisements looking for HIV seronegative volunteers, and were recruited into the SCOPE cohort [9].

For all of the cohorts involved, participants were selected in consecutive manner, and began when funding was made available for these studies, and stopped when funding was no longer available. Hence, there were no a priori criteria used to select the specimens.

### CMV-Specific T Cell Responses

All T cell studies were performed using freshly collected peripheral blood. The same method and the same laboratory were used for all study participants [36]. For both pp65 and IE responses, a pool of 15 amino acid peptides (each overlapping by 11 amino acids) from each protein was synthesized. Fresh whole blood was then stimulated with these peptide pools in the presence of brefeldin A, an inhibitor of intracellular transport that blocks the extracellular secretion of cytokines. Non-stimulated and superantigen staphylococcal enterotoxin B (SEB)-stimulated samples were used as negative and positive controls, respectively. Cells were then fixed and permeabilized before incubation with fluorescein isothiocyanate (FITC)-conjugated anti-IFN-γ, phycoerythrin (PE)–conjugated anti-CD69, phycoerythrin–cyanin red 5.1 (PC5)–conjugated anti-CD4, and allophycocyanin (APC)-conjugated anti-CD3. The fraction of activated, cytokine-secreting (CD69+IFN-γ+) CD4+ and CD8+ T lymphocytes was determined by flow cytometry using a Becton Dickinson FACS Calibur. The lymphocyte gate was determined by forward and side scatter.

In our primary analysis, we focused only on those cells that stained brightly for IFN-γ (to reduce the number of false positive responses). These “IFN-γ bright” events were defined as CD69+ IFN-γ+ events that fell 3 decades above the IFN-γ-negative population in non-stimulated controls, as previously described [37]. All measurements were background corrected. We also measured IL2 and IFN-γ production in response to pp65 and IE in a subset of subjects, standard gating was used for both cytokines.

### Statistical Analylsis

Wilcoxon rank sum tests were used to compare the percent CMV-specific T cells between groups. Unadjusted associations between continuous variables were assessed with Spearman's rank order correlation coefficients. Adjusted associations were assessed with linear regression, transforming continuous variables and calculating standard errors with heteroskedasticity-consistent covariance matrix estimators when necessary to satisfy model assumptions [38]. Age, gender, CD4+ T cell count, plasma HIV RNA level, and disease stage (acute versus chronic, treated versus not treated) were all considered potential confounders in multivariable models, but were removed in a stepwise manner if their inclusion changed the beta coefficient of the primary predictor by <10%. The validity of model assumptions and the role of influential observations were assessed for each regression analysis.

## Results

A total of 685 unique individuals were included in this study (Table 1). Eighty-three of these were HIV seronegative and most of them were men (n = 75) who were CMV seropositive (n = 60). The remaining 602 subjects represented a broad spectrum of treated and untreated HIV infection: 77 were antiretroviral-untreated and recently infected (estimated infection duration of less than 180 days); 176 were antiretroviral-untreated and chronically infected (median CD4 count of 525 cells/mm^3^); 203 were antiretroviral-treated and had undetectable plasma HIV RNA levels (median CD4 of 503 cells/mm^3^); and 146 were antiretroviral-treated had a detectable plasma HIV RNA level (median CD4 count of 277 cells/mm^3^). Of the 332 HIV-infected participants whose CMV antibody status was tested, only 4 (1.2%) were CMV seronegative. The median ages of the HIV-seronegative and HIV-seropositive subjects were 40 and 44 years.

**Table 1:** Baseline characteristics.

	HIV− CMV−	HIV− CMV+	Acute/recent HIV infection (<180 days)	Chronic, untreated HIV infection	Antiretroviral-treated (undetectable viral load)	Antiretroviral-treated (detectable viral load)
**Number**	23	60	77	176	203	146
**Age (years)**	40 (30–46)	39.2 (34.5–45.8)	35.9 (30.5–41.1)	43.8 (38.1–49.8)	45.1 (39.3–51.9)	46.7 (42.3–52.4)
**Gender (% female)**	13%	8%	4%	22%	8%	15%
**CD4+ T cell count (cells/mm^3^)**	NA	948 (689–1058)	578 (480–722)	525 (349–764)	503.5 (329–752)	278 (171–437)
**CD8+ T cell count (cells/mm^3^)**	NA	491 (324–647)	850 (630–1139)	942 (692–1279)	889 (619–1182)	1067 (741–1476)
**Plasma HIV RNA (log_10_ copies/mL)**	NA	NA	4.55 (3.83–4.93)	2.92 (1.88–4.36)	1.88 (1.88–1.88)	3.53 (2.64–4.33)

**NOTE.** Median values and interquartile ranges are shown unless otherwise noted.

### CMV Specific T Cell Responses in CMV-Seropositive and Seronegative Individuals

**Figure 1:**
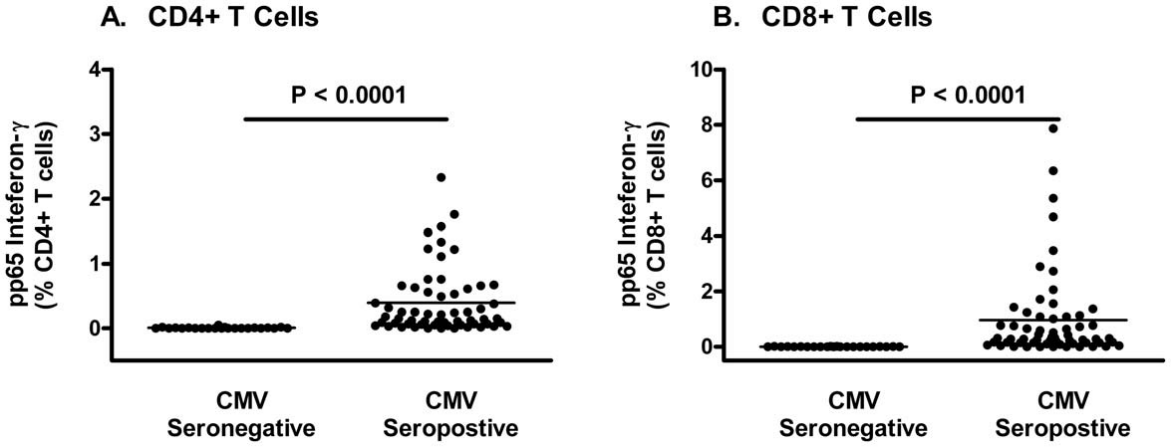
The distribution of percent of CMV-specific T cells in CMV-seronegative and CMV-seropositive, HIV-uninfected subjects.

To understand the relationship between CMV-specific T cell responses and CMV serology, the frequency of pp65-specific IFN-γ bright CD4+ and CD8+ T cell responses was compared between HIV seronegative/CMV-seronegative (n = 23) subjects and the HIV seronegative/CMV-seropositive subjects (n = 60). No CMV-seronegative individual had detectable pp65-specific CD4+ and CD8+ T cell responses above 0.05%, while the vast majority of CMV-seropositive individuals had detectable responses above these levels (Table 2 and Figure 1). The differences were significant for both CD4+ and CD8+ CMV-specific T cells (p<0.0001 for each pair-wise comparison between CMV-seronegative and seropositive groups). Each of the four HIV-seropositive, CMV-seronegative participants had no or only very low-levels of pp65-specific T cell responses.

**Table 2:** CMV-specific T cell responses in HIV uninfected and infected adults.

	HIV− CMV−	HIV− CMV+	Acute/recent HIV infection (<180 days)	Chronic, untreated HIV infection	Antiretroviral-treated (undetectable viral load)	Antiretroviral-treated (detectable viral load)
**pp65-specific interferon-γ-bright CD4+ T cells (%)**	0.00 (0–0.01)	0.15 (0.06–0.61)	0.22 (0.11–0.49)	0.48 (0.13–1.17)	0.25 (0.1–0.74)	0.19 (0.05–0.58)
**pp65-specific interferon-γ-bright CD8+ T cells (%)**	0.00 (0–0.01)	0.31 (0.14–1.09)	0.67 (0.28–1.43)	0.93 (0.27–2.2)	1.67 (0.57–3.46)	1.38 (0.55–3.37)
**IE-specific interferon-γ-bright CD4+ T cells (%)**	0.00 (0–0.01)	0.02 (0–0.05)		0.02 (0–0.07)	0.04 (0.01–0.1)	0.03 (0–0.06)
**IE-specific interferon-γ-bright CD8+ T cells (%)**	0.00 (0–0)	0.13 (0.02–0.86)		0.11 (0.03–0.39)	0.62 (0.20–2.5)	0.20 (0.04–0.85)
**pp65- and IE- interferon-γ/IL2 CD4+ T cells (%)**	0.06 (0.02–0.14)	0.24 (0.13–0.8)		0.52 (0.23–1.58)	0.70 (0.33–1.57)	0.36 (0.24–1.7)
**pp65- and IE- interferon-γ/IL2 CD8+ T cells (%)**	0.03 (0.01–0.06)	2.06 (0.81–3.51)		1.73 (0.82–3.17)	4.67 (2.36–9.11)	2.97 (1.05–4.37)

NOTE. Median values and interquartile ranges are shown. All subjects contributed pp65-specific IFN-γ-bright data (n = 685), while only a subset contributed pp65 and IE data (see text). IE data were not available from the recently infected subjects. The combined pp65 and IE interferon-γ/IL2 data reflect the sum of all possible response (IFN-γ alone, IL2 alone and IFN-γ/IL2) to each of the CMV protein.

For all of the following analyses, we restricted the HIV-seronegative subset to those who were known to be CMV-seropositive. Since 98.8% of our HIV-infected participants with CMV antibody data were seropositive, we included in the following analysis all HIV subjects who were known to be CMV-seropositive, as well as the additional subjects for whom the CMV sero-status was not known. Notably, the vast majority of subjects without CMV antibody data had clear evidence of CMV infection based on their pp65-specific T cell responses. For example, all confirmed CMV-seronegative subjects in our study had a background corrected pp65-specific CD8+ T cell interferon-γ response of <0.03%, while only 16 of the 277 evaluable subjects without CMV antibody data had CD8+ T cell responses below this level (and 8 of these 16 had much stronger pp65 specific CD4+ T cell response than the confirmed CMV-seronegative subsets).

### Impact of Recent and Chronic Untreated HIV Infection on CMV Specific T Cell Responses

**Figure 2:**
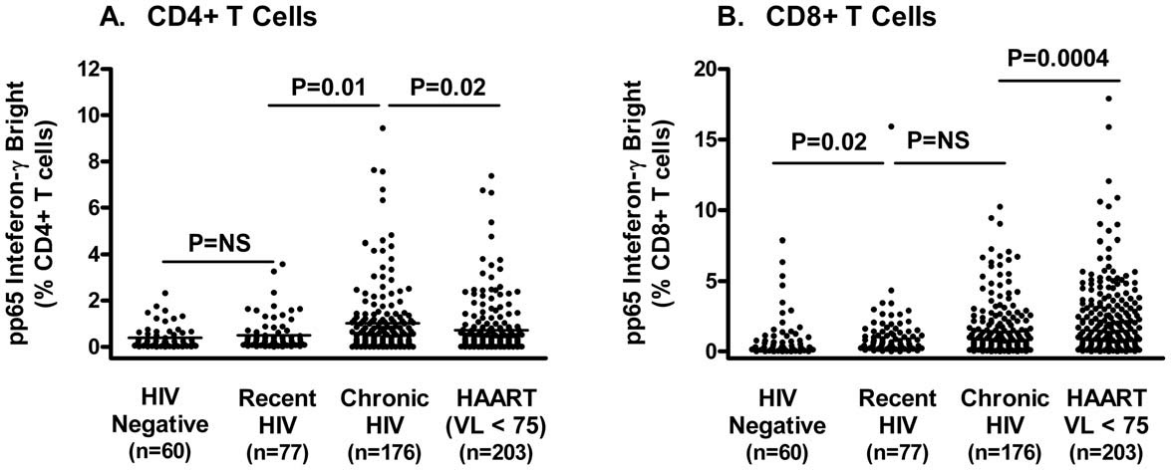
The distribution of pp65 IFN-γ-bright CD4+ and CD8+ T cell responses (background corrected) in four unique groups: (1) HIV-seronegative, CMV-seropositive, (2) acute and recent untreated HIV infection, (3) established chronic untreated HIV infection, and (4) antiretroviral-treated infection with undetectable plasma HIV RNA levels.

We next addressed the question of whether HIV infection is associated with an altered CMV-specific T cells response. As compared to the CMV-seropositive, HIV-seronegative group, untreated participants with a recent HIV infection (n = 77) had a higher median frequency of pp65-specifc IFN-γ bright CD8+ T cells (0.31% vs. 0.67%, respectively, P = 0.02) and a similar median frequency of pp65-specifc IFN-γ bright CD4+ T cells (0.15% vs. 0.22%, P = 0.15; Figure 2).

The 178 untreated, chronically HIV-infected participants had even higher median frequencies of pp65-specifc IFN-γ bright CD8+ T cells (median 0.93%) and pp65-specifc IFN-γ bright CD4+ T cells (median 0.48%) (P<0.001 for the comparison of both CD8+ and CD4+ T cell responses between the HIV-seronegative subjects and the chronically infected subjects).

The absolute number of pp65-specific CD8+ T cells was significantly elevated in both HIV-seropositive groups versus the HIV-seronegative group (P<0.001 for each pairwise comparison; data not shown). The absolute number of pp65-specific CD4+ T cells was also slightly elevated among the two HIV-seropositive groups compared to the HIV-seronegative group.

### CMV-Specific T Cell Responses Are Higher in Antiretroviral Treated Persons

We next studied 203 antiretroviral-treated subjects who had undetectable plasma HIV RNA levels. The median pp65-specific IFN-γ-bright CD8+ T cell response in this group was 1.7% (IQR 0.6 to 3.5%) and the median CD4+ response was 0.25% (IQR 0.10 to 0.74%). These responses were higher than those observed in the HIV-seronegative, CMV-seropositive group (P<0.0001 for CD8+ T cell responses between the two groups and P = 0.06 for the CD4+ T cell responses between the two groups). These responses were also higher than those observed in the group of chronic untreated participants (P = 0.0004 for CD8+ T cell responses between the two groups and P = 0.02 for the CD4+ T cell responses between the two groups).

We also studied 146 antiretroviral-treated subjects had detectable viremia (the majority of these subjects were known to have an extensive history of drug-resistant viremia, as previously described [37]). The pp65-specific responses in this group were comparable to those observed in the treated subjects who had undetectable viremia (median values of 1.38% and 0.19% pp65-specific CD8+ and CD4+ T cell response, respectively).

### Impact of Disease State on IE-Specific T Cell Responses

The intermediate early (IE) protein-specific T cells responses were obtained all 83 HIV-seronegative individuals (60 of whom were CMV seropositive) as well as on 20 subjects who had chronic untreated HIV infection and 60 subjects who were on antiretroviral therapy and had an undetectable plasma HIV RNA level. As expected, the IE-specific responses were higher in the HIV-seronegative, CMV-seropositive subjects than in the HIV-seronegative, CMV-seronegative subjects (P<0.0001 for both CD4 and CD8 responses).

**Figure 3:**
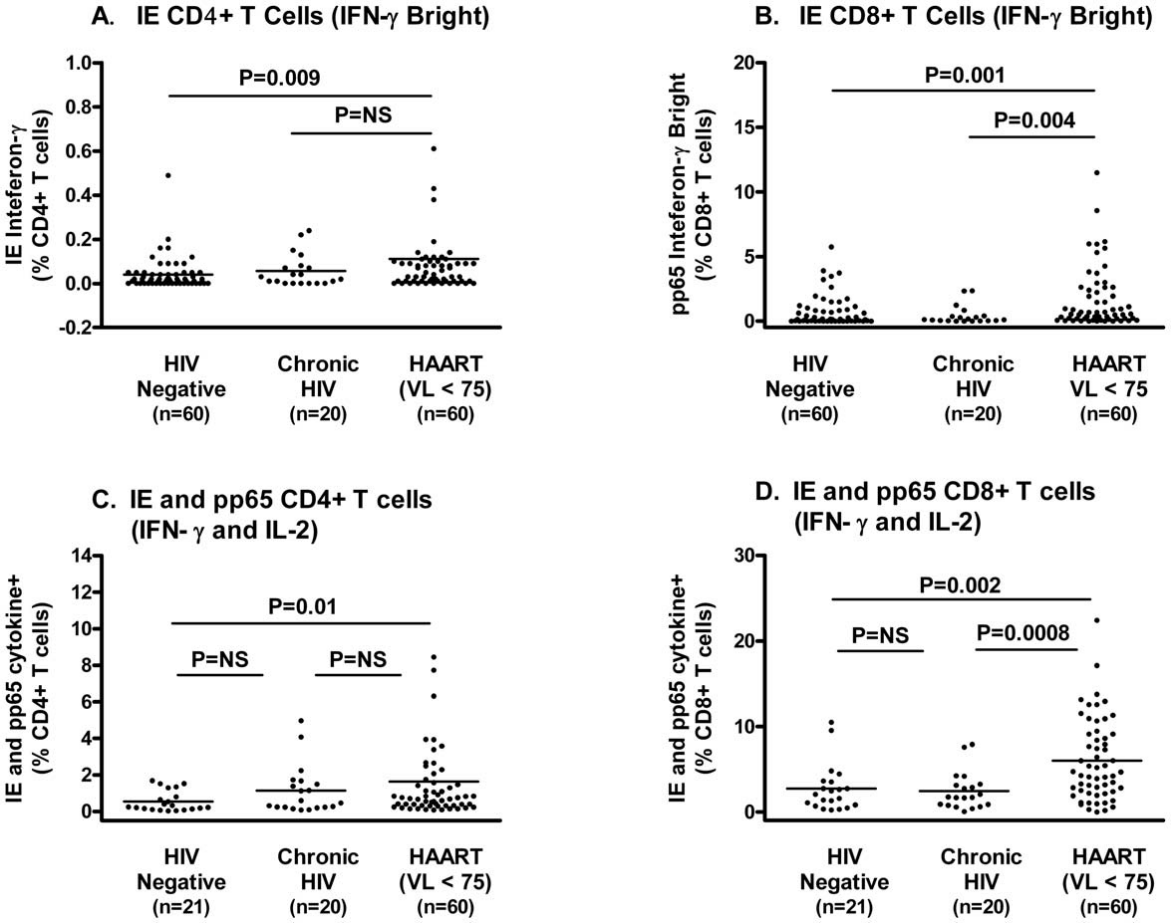
The distribution of the CMV-specific CD4+ and CD8+ T cell responses (background corrected) in three unique groups: (1) HIV-seronegative, CMV-seropositive, (2) established chronic untreated HIV infection, and (3) antiretroviral-treated infection with undetectable plasma HIV RNA levels. The IE IFN-γ-bright levels are shown in panel A (CD4+ T cells) and panel B (CD8+ T cells). The combined pp65 and IE IFN-γ/IL2 data are shown in panels C (CD4+ T cells) and panel D (CD8+ T cells). The data in panels C and D reflect the sum of all possible response (IFN-γ alone, IL2 alone, or IFN-γ/IL2) to each of the CMV proteins. Standard gating was used for dual cytokine data.

The effect of antiretroviral-treated HIV on IE-specific response was similar to that observed with pp65 (Figures 3A and 3B). The median proportion of IE-specific IFN-γ-bright CD4+ T cells was 0.02% in the HIV-seronegative/CMV-seropositive, 0.03% in the chronic, untreated subjects, and 0.05% in antiretroviral treated, fully suppressed subjects (P = 0.009 for HIV negative vs. treated HIV). The median proportion of IE-specific IFN-γ-bright CD8+ T cells was 0.13% in the HIV-seronegative/CMV-seropositive subjects, 0.11% in the chronically infected, untreated subjects, and 0.62% in the antiretroviral treated, fully suppressed subjects (P = 0.001 for HIV seronegative vs. treated HIV).

### Impact of Disease State on pp65- and IE-Specific IFN-γ and IL2 T Cell Responses

Multi-functional (IFN-γ and IL2) responses to pp65 and IE were also measured in a subset of subjects (21 subjects who were CMV-seropositive/HIV-seronegative, 20 subjects who had chronic untreated HIV infection and 60 subjects who were on antiretroviral therapy and had undetectable plasma HIV RNA levels) (Figure 3). In general, the pp65-specific responses were higher than the IE-specific responses, as previously shown [31]. This was true for both CD4+ and CD8+ T cell populations. Also, most responding cells made either IFN-γ alone or IFN-γ and IL2 (cells producing IL2 only were rare). The only exception was the pp65-specific CD8+ T cell population, where the vast majority of cells made IFN-γ alone.

To enumerate as many of CMV-specific cells as possible, we combined the all of the pp65 and IE specific responses (i.e, IFN-γ only, IL2 only, IFN-γ and IL2 populations were summed) (Figures 3). The mean combined CD8+ T cells responses for these two cytokines against these two CMV proteins were 2.7, 2.4, and 6.0% in HIV-seronegative/CMV-seropositive subjects, chronic untreated subjects and long-term treated groups, respectively (P = 0.002 for HIV-negative vs. antiretroviral-treated, and P = 0.0008 for chronic untreated vs. antiretroviral treated).

### CMV-Specific T Cell Responses Are Elevated Before the Onset of Immunodeficiency

Theoretically, HIV-associated immunodeficiency may result in increased levels of CMV replication and this in turn could induce increased levels of CMV-specific T cell responses. To determine if CMV-specific T cell responses were elevated before the onset of immunodeficiency, we identified those study participants who were antiretroviral-untreated and had at the time of the analysis a peripheral CD4+ T cell count greater than 500 cells/mm^3^ (n = 146). The pp65-specific CD4+ T cell response in these presumably immunocompetent individuals was 0.32%, which was significantly higher than that found in CMV-seropositive, HIV-seronegative adults (median 0.15%, P = 0.009). The pp65-specific CD8+ T cell response was 0.62%, which was also higher than that in CMV-seropositive HIV-seronegative adults (median 0.31%, P = 0.02).

### Predictors of CMV-Specific T Cell Responses in Untreated and Treated HIV Infection

We next assessed the factors associated with CMV-specific responses, focusing on the pp65 IFN-γ bright data as this was the only outcome available on the entire cohort. In unadjusted analysis, the most consistent predictor of both the pp65-specific CD4+ and CD8+ T cell response was age, although the strength of these associations was generally weak. For example, amongst the long-term antiretroviral treated patients with undetectable viral loads, age was positively correlated with both pp65-specific CD4+ T cells responses (rho = 0.26, P = 0.0001) and CD8+ T cell responses (rho = 0.24, P = 0.0004).

The peripheral CD4+ T cell count was negatively associated with the pp65-specific T cell responses, but again the associations were weak. Amongst the untreated cohort, CD4+ T cell counts were negatively associated with the pp65-specific CD8+ T cell responses (rho = −0.27; P = 0.0002). Weak, marginally significant negative associations were observed among the treated individuals who had undetectable viremia. There were no consistent associations between plasma HIV RNA levels and pp65-specific T cell responses.

We next performed multivariable linear regression to determine what role age and other factors may have had in our comparisons between groups (Tables 3–6). After adjustment for age, CD4+ T cell count, and gender, the long-term antiretroviral-treated subjects with undetectable viremia had a higher proportion of pp65-specific CD8+ T cell responses than the HIV-seronegative, CMV-seropositive subjects (P = 0.009). After controlling for these same factors, there was no difference in the pp65-specific CD4+ T cell responses (P = 0.46). The differences in pp65-specific T cell responses between the antiretroviral-treated subjects (with undetectable viremia) and the untreated subjects remained significant after controlling for age, gender and CD4+ T cell count (P = 0.008 for adjusted comparison of pp65-specific CD8+ T cells response and P = 0.005 for the adjusted comparison of pp65-specific CD4+ T cell responses).

**Table 3:** Percentage of pp65-specific interferon-γ+ CD8+ T cells in antiretroviral treated subjects with undetectable viral loads and HIV seronegative subjects.

	Coefficient	P	95% CI	
HIV	0.95	0.009	0.24	1.66
Age (per 10 years)	0.57	0.002	0.21	0.94
Female	−0.19	0.83	−1.95	1.57
CD4 (per 100 cells/µL	−0.010	0.005	−0.02	−0.003
Constant	−0.50	0.54	−2.10	1.10

**Table 4 pone-0008886-t004:** Percentage of pp65-specific interferon-γ + CD4+ T cells in antiretroviral treated subjects with undetectable viral loads and HIV seronegative subjects.

	Coefficient	P	95% CI	
HIV	−0.12	0.46	−0.43	0.20
Age (per 10 years)	0.32	0.004	0.10	0.53
Female	−0.44	0.037	−0.86	−0.03
CD4 (per 100 cells/µL	−0.005	0.009	−0.009	−0.001
Constant	−0.29	0.54	−1.22	0.64

**Table 5 pone-0008886-t005:** Percentage of pp65-specific interferon-γ+ CD8+ T cells in antiretroviral treated subjects with undetectable viral loads and in untreated HIV infected subjects.

	Coefficient	P>t	95% CI	
Treated versus untreated	0.66	0.008	0.17	1.15
Age (per 10 years)	0.58	0.000	0. 32	0.85
Female	−0.39	0.29	−1.11	0.34
CD4 (per 100 cells/µL	−0.009	0.004	−0.015	0.003
Constant	−0.32	0.59	−1.52	0.87

**Table 6:** Percentage of pp65-specific interferon-γ+ CD4+ T cells in antiretroviral treated subjects with undetectable viral loads and in untreated HIV infected subjects.

	Coefficient	P>t	95% CI	
Treated versus untreated	−0.40	0.005	−0.68	−0.12
Age (per 10 years)	0.40	0.000	0.21	0.59
Female	−0.41	0.02	−0.75	−0.064
CD4 (per 100 cells/µL	−0.003	0.095	−0.007	0.0005
Constant	−0.48	0.29	−1.38	0.41

## Discussion

Using our large cohort of HIV -seropositive persons in various stages of HIV infection, as well as a large cohort of HIV-seronegative persons, we investigated the impact of HIV and its treatment on CMV-specific T cell responses. Our work was driven in part by emerging data that chronic CMV infection can have profound detrimental effects on immune function as people age [39], [40]. When all data across all cohorts are considered, a complex but consistent story emerges. Early HIV infection was associated with higher frequencies of pp65-specific CD8+ T cell responses (as compared to HIV seronegatives), confirming previous reports [41], [42]. This occurred before the onset of measurable immunodeficiency. CMV-specific CD8+ T cell responses were higher in chronic untreated HIV infection compared to recent HIV infection. Surprisingly, these responses were even higher among antiretroviral-treated individuals (on average, 6% of the peripheral CD8+ T cells from these subjects produced IFN-γ and/or IL-2 in response to CMV, a finding even more remarkable when considering that we measured responses against only two of the over 200 CMV-encoded proteins). The CMV-specific CD4+ T cell responses follow similar trends. These data indicate that CMV infection has a profound effect on the adaptive immune response during untreated HIV disease and this effect is accentuated by long-term administration of combination antiretroviral therapy.

It is now widely accepted that chronic CMV infection has profound effects on the immune system in the very old. As compared to elderly CMV seronegative adults, those who are CMV seropositive have lower CD4/CD8 ratios, lower naïve T cell numbers, higher frequencies of well differentiated CD8+CD28− memory cells and perhaps a smaller T cell repertoire and [22], [25]. Based on extensive experimental data, a theoretical model has emerged that could provide an mechanistic basis for these observations [39], [40], [43], [44], [45], [46]. Specifically, chronic viral infections that establish latency (e. g., the herpes viruses) cause persistent antigenic stimulation to T cells. These T cells continually expand in response to this proinflammatory environment, eventually forming a large population of well-differentiated, apoptosis-resistant, virus-specific CD8+ T cells with limited proliferative potential. These cells take up “immunologic space”, preventing the generation of new naïve and central memory cells (a process that may be accentuated by the age-related decline in thymic function [16], [47]). Collectively, these change result in accelerated immunologic aging, or “immunosenescence”, which in turn contributes to age-associated morbidities. Theoretically, HIV-associated inflammation and HIV-associated thymic dysfunction may cause many of these same changes to the adaptive immune system [43], [48].

Although the rates of CMV end organ disease have dropped dramatically since the introduction of combination antiretroviral therapy, a number of studies indicate that CMV seropositivity continues to be associated with significant morbidity and mortality in the setting of chronic HIV infection. Most notably, Griffiths and colleagues found that among patients with advanced HIV disease (most of whom were on antiretroviral therapy), plasma CMV DNA levels was strongly predictive of mortality [7]. Several mechanisms have been suggested, including the positive effect that CMV and other herpes viruses may have on HIV replication [49], [50], [51]. We favor an indirect effect of CMV on outcome via the strong proinflammatory role that CMV appears to have *in vivo* [28]. Given the well-accepted role of inflammation as key component of HIV associated immunopathogenesis [52], [53], [54], [55], [56], it is reasonable to assume that CMV-associated inflammation would have a largely detrimental effect on disease outcomes.

The persistent CMV associated inflammatory response outlined here is also notable for its magnitude. The levels of pp65-specific interferon-gamma producing CD8+ T cells in our antiretroviral-treated patients was comparable to that observed in very old HIV seronegative persons (>85 years of age) [57]. On average, 6% of the circulating CD8+ T cells in antiretroviral-treated patients recognize either pp65 or IE peptides as defined by the production of interferon-γ and/or IL2. The total CMV specific T cell response is almost certainly much greater since pp65 and IE reflect only one of over 200 open reading frames in CMV, many of which are known to be immunogenic [28]. Also, many antigen-specific T cells do not make IFN-γ and/or IL2 in response to *ex-vivo* peptide stimulation. Assuming that the pp65- and IE- specific IFN-γ/IL2 responses makes up only a small fraction of the total CMV-specific T cell responses (a reasonable assumption given that this has been clearly demonstrated in HIV seronegative individuals [28]), then the overall CMV-specific inflammatory response in antiretroviral-treated HIV disease is likely to be enormous (since CMV-specific responses were at least twice as high in our treated HIV infected patients than in the HIV seronegative subjects, and since one comprehensive study of healthy CMV-seropositive adults found that on average 10% of the T cell population was CMV-specific [28], then it seems likely that approximately 20% of all circulating CD8+ T cells in antiretroviral-treated patients are directed at this single virus).

In summary, heightened CD8+ T cell activity directed against CMV is observed in early HIV infection, persists at higher levels in chronic untreated HIV infection, and is accentuated even further upon administration of effective combination antiretroviral therapy. Possible mechanisms for the very high frequencies of CMV-specific T cells in antiretroviral-treated HIV disease include sub-clinical CMV replication and/or a dysregulated and heightened immunologic response to a normal level of CMV replication (perhaps as a consequence of loss of certain immunoregulatory cells). The recent observation that CMV-specific CD4+ T cells secrete large amounts of chemokines that protect these cells from HIV entry may also be a contributing factor [58]. Regardless of mechanism, this disproportionate CMV cellular reactivity during antiretroviral therapy is likely to be associated with harm, as we have recently demonstrated [9] and has been extensively demonstrated in the very old. Future longitudinal studies to determine whether CMV drives early immunosenescence during HIV disease are warranted.
